# Classes 1 and 2 integrons in faecal *Escherichia coli* strains isolated from mother-child pairs in Nigeria

**DOI:** 10.1371/journal.pone.0183383

**Published:** 2017-08-22

**Authors:** Babatunde W. Odetoyin, Amy S. Labar, Adebayo Lamikanra, Aaron O. Aboderin, Iruka N. Okeke

**Affiliations:** 1 Department of Medical Microbiology and Parasitology, Obafemi Awolowo University, Ile-Ife, Osun State, Nigeria; 2 Department of Biology, Haverford College, Haverford, Pennsylvania, United States of America; 3 Department of Pharmaceutics, Faculty of Pharmacy, Obafemi Awolowo University, Ile-Ife, Osun State, Nigeria; 4 Faculty of Pharmacy, University of Ibadan, Ibadan, Oyo State, Nigeria; Leibniz-Institute DSMZ, GERMANY

## Abstract

**Background:**

Antimicrobial resistance among enteric bacteria in Africa is increasingly mediated by integrons on horizontally acquired genetic elements. There have been recent reports of such elements in invasive pathogens across Africa, but very little is known about the faecal reservoir of integron-borne genes.

**Methods and findings:**

We screened 1098 faecal *Escherichia coli* isolates from 134 mother-child pairs for integron cassettes by PCR using primers that anneal to the 5’ and 3’ conserved ends of the cassette regions and for plasmid replicons. Genetic relatedness of isolates was determined by flagellin and multi-locus sequence typing. Integron cassettes were amplified in 410 (37.5%) isolates and were significantly associated with resistance to trimethoprim and multiple resistance. Ten cassette combinations were found in class 1 and two in class 2 integrons. The most common class 1 cassette configurations were single *aadA1* (23.4%), *dfrA7* (18.3%) and *dfrA5* (14.4%). Class 2 cassette configurations were all either *dfrA1-satI-aadA1* (n = 31, 7.6%) or *dfrA1-satI* (n = 13, 3.2%). A *dfr* cassette was detected in 294 (31.1%) of trimethoprim resistant strains and an *aadA* cassette in 242 (23%) of streptomycin resistant strains. Strains bearing integrons carried a wide range of plasmid replicons of which FIB/Y (n = 169; 41.2%) was the most frequently detected. Nine isolates from five different individuals carried the *dfrA17-aadA5*-bearing ST69 clonal group A (CGA). The same integron cassette combination was identified from multiple distinct isolates within the same host and between four mother-child pairs.

**Conclusions:**

Integrons are important determinants of resistance in faecal *E*. *coli*. Plasmids in integron-containing strains may contribute to dispersing resistance genes. There is a need for improved surveillance for resistance and its mechanisms of dissemination and persistence and mobility of resistance genes in the community and clinical settings.

## Introduction

The level of antibiotic resistance among pathogenic and commensal bacteria has steadily increased and has become a global health concern [1]. Much of the problem has been attributed to mobile genetic elements such as plasmids and transposons [2]. Integrons are genetic elements that also contribute to the prevalence and horizontal transmission of antibiotic resistance [3,4,5]. Integrons are genetic elements with a site-specific recombination system for capturing, expressing and exchanging gene cassettes [6]. They are characterized by conserved features, namely an *intI* gene encoding an integrase, a recombination site (*attI)*, a promoter (P) and the ability to integrate gene cassettes comprise of a single open reading frame (orf) and a specific recombination site, *attC* [4,7]. Intracellularly, gene cassettes exist either in a linear form inserted into an integron or as a free circular cassette that is not dependent on an integron [7, 8]. At least nine classes of integrons have been described and class 1and 2 integrons are the most predominantly associated with antibiotic resistance in clinical isolates [9]. The capture of resistance genes is especially important when these integrons are disseminated by broad-host-range conjugative plasmids or transposons.

So far, more than 8000 gene cassette arrays have been identified in class 1 integrons (http://integrall.bio.ua.pt/). Yet, less than 10 array compositions are commonly reported [9,10,11]. These commonly reported arrays are found in a wide variety of hosts and environments, highlighting a high level of horizontal dissemination of these elements among bacterial populations and species. Clonal expansion also contributes to the current prevalence of inter-regional spread of integron-carrying bacterial species. However, the extent to which each contributes is unknown [12]. In Nigeria, there have been reports of integrons in strains isolated from the clinical and environmental settings [13,14,15,16]. However, very little is known about the faecal reservoir of integron-borne genes. In this study, we investigated prevalence of classes 1 and 2 integrons, their association with antimicrobial resistance and how resistance cassettes are disseminated in faecal, *Escherichia coli* strains isolated from children with diarrhoea and their apparently healthy mothers.,

## Materials and methods

### Ethics statement, study population and sample collection

The investigation protocols used in this study were approved by the Ethics and Research Committee of Obafemi Awolowo University Teaching Hospitals Complex (OAUTHC), Ile-Ife. All the participants gave verbal informed consent after explaining the purpose and procedures of the study to them. Parents, guardians or caretakers consented on behalf of the children before recruitment at the State Hospital Oke-Ogbo, Ile-Ife, Osun State, between February 2008 and February 2011.

A total of 134 children up to five years of age with diarrhoea of not more than two weeks duration, paired with their mothers, was included in this study. Children without diarrhoea and those who had taken antibiotics before coming to the hospital were excluded. Consenting mothers and their children were requested to produce stool samples in sterile universal bottles. All samples were transported to the laboratory for processing.

### Isolation and identification of *Escherichia coli* strains

All faecal samples were inoculated onto Eosin Methylene Blue (EMB) agar plates (Oxoid Ltd., Basingstoke, Hampshire, England) and incubated for 24 hours aerobically at 37°C. From each sample, up to five morphologically distinct colonies typical of *E*. *coli* were selected and identified by standard biochemical testing [17].

### DNA extraction

All isolates were grown overnight in 5 ml of peptone broth (Oxoid, England). A 1 ml aliquot of the culture was centrifuged at 10,000 rpm for two minutes in a microcentrifuge (BioRad, USA). DNA was extracted from each isolate using the Promega Wizard genomic extraction kit (Promega, corporation, Madison, USA) according to the manufacturer’s instructions.

### Antimicrobial susceptibility testing

The antimicrobial susceptibility testing for each isolate was performed by the Kirby-Bauer disc diffusion technique on Mueller-Hinton agar (CM0337) (Oxoid Ltd., Basingstoke, Hampshire, England). Antibiotics tested were ampicillin (AMP) (l0μg), streptomycin (S10) (l0μg), ciprofloxacin (CIP) (5μg), nalidixic acid (NAL) (30μg), tetracycline (TET) (30μg), chloramphenicol (C30) (30 μg), sulphonamides (SUL) (300μg) and trimethoprim (W) (5 μg) (Remel, U.S.A). The inoculated plates were incubated at 37°C for 24 hours. Interpretation of the diameters of the zones of inhibition was made according to the guidelines of the CLSI [18].

### Detection of class 1 and 2 integrons

All the isolates were screened for class 1 and 2 integrons by polymerase chain reaction (PCR). Class 1 integron was detected and amplified by Levesque 5CS and 3CS primers which bind the 5' and 3' conserved ends respectively [19]. Class 2 integron was detected and amplified by White hep74 and White hep51 to hybridize *attI2* and *orfX* respectively [20] (S1 Table).

The following PCR cycle, adapted from proposed by Lévesque *et al*. (1995, with modifications) was used to amplify the variable regions of both class 1 and class 2 integrons. following a 2 minute hot start at 94°C, 40 cycles of denaturation 94°C for 30s, annealing 57°C for 30s, extension 72°C for 1 min per kb were performed. *E*. *coli* strains 042 (carrying a class 1 integron) and 17–2 (carrying a class 2 integron) were used as positive controls in the integron PCRs and *E*. *coli* K-12 strain DH5α, lacking integrons, was used as a negative control. 5μl of each PCR reaction was electrophoresed on a 1% agarose gel in 1X Tris Acetate EDTA buffer. Gels were stained with ethidium bromide for 15 minutes, destained in distilled water for 30 minutes, and visualized under ultraviolet (UV) light.

### Identification of resistance cassettes and cassette combinations in integrons

Unique resistance cassettes and cassette combinations within class 1 and 2 integrons were delineated by performing restriction fragment length polymorphism (RFLP) with *Mbo*1 (Biolab, England) and *Alu*1 (Biolab, England) as previously described [21]. Up to three amplicons from each unique profile were ligated into a pGEMT-Easy vector (Promega, USA) and sequenced. The identity of contained cassettes was determined using BLAST [22].

### Plasmid replicon typing

Two multiplex panels comprised of 11 primer pairs were used to identify plasmid replicons by PCR as described by Carattoli *et al* [23]. Strains carrying well-characterized plasmids pMAR-7, pB171, pHCM1 and pED204 were used as controls [24,25,26,27].

### Flagellin typing

The entire coding sequence of the variable chromosomal gene *fliC* was amplified by PCR using the primers F-FLIC (5'-ATG GCA CAA GTA ATT AAT AAC CAA C-3') and R-FLIC (5' CTA ACC CTG CAG CAG AGA CA-3') [28]. The cycling conditions were 30s at 95°C, 1 min at 60°C, and 2 min at 72°C for 35 cycles. PCR product was digested with *Rsal* (Promega, USA) as described by Fields *et al*. (1997) [29]. The restriction profiles were compared after electrophoresis on 1.5% agarose gel. The H-type of flagellin RFLPs of interest was determined by sequencing and BLAST analysis.

### Multi locus sequence typing (MLST)

Greater resolution of similarity between chromosomes of selected isolates was provided by multilocus sequence typing. This was done by amplifying seven housekeeping genes namely adenylate kinase (*adk*), fumarate hydratase (*fumC*), gyrase B *(gyrB*), isocitrate dehydrogenase (*icd*), malate dehydrogenase (*mdh*), adenylosuccinate synthetase (*purA*) and recombinase A (*recA*) with primers described by Wirth *et al* [30] (S2 Table). Cycling conditions were as follows: 95°C for 2 minutes, followed by 35 cycles of 94°C for 1 minute, 56°C for 1 minute and 72°C for 1 minute. A final, 3 minute elongation step was performed at 72°C. Amplified DNA products of the housekeeping genes were sequenced and alleles were assigned by using an online *E*. *coli* MLST database at http://www.mlst.net.

### Statistical analysis

The Chi-square (χ2) and Fischer’s exact tests (two-tailed) of R statistical software package (version 3.3.0) were used to determine the statistical significance of the data. Pearson Product Moment Correlation Coefficient, r, calculated using SPSS (SPSS, Inc. Chicago, Illinois) was used to identify significant correlations. All reported p-values were two-sided and a p-value of less than or equal to 0.05 was considered to be statistically significant.

## Results

### Subjects and faecal *Escherichia coli* isolates

A total of 1098 *Escherichia coli* strains was isolated from the stool samples of 134 mother and child pairs recruited into the study. These comprised of 542 isolates from children aged up to 60 months (13.36±2.12) and 556 isolates from their mothers’ ages of 15–46 years (25.88 ±7.07).

### Antimicrobial resistance rates in the faecal *Escherichia coli* isolates

As shown in Table 1, the majority of the *E*. *coli* isolates from both mothers and their children were resistant to streptomycin (n = 1050, 95.8%), sulphonamide (n = 1038, 94.2%), ampicillin (n = 1014, 92.5%), tetracycline (n = 1025, 93.3%) and trimethoprim (n = 947, 85.9%) except to ciprofloxacin (n = 94, 8.4%). Isolates that were resistant to ciprofloxacin (χ^2^ = 6.498; p = 0.009) were more frequently isolated from healthy mothers than their children. The differences in resistance to other antimicrobials were not significantly different between isolates obtained from children and those from mothers.

**Table 1:** Antimicrobial resistance patterns and integron contents of faecal *Escherichia coli* isolates from mother and child pairs.

Antimicrobial Agents	Diarrheic Children (%) (n = 542)	Apparently Healthy Mothers (%) (n = 556)	P-value^a^	Integron- Positive (%) (n = 410)	Integron- Negative (%) (n = 688)	P-value	Total (%) N = 1098
Tetracycline	511 (94.3)	514 (92.4)	0.223	385 (93.9)	640 (93)	0.674	1025 (93.3)
Trimethoprim	462 (85.2)	485 (87.2)	0.338	381 (92.9)	566 (82.3)	0.004^b^	947 (85.9)
Sulphonamide	520 (95.9)	518 (93.2)	0.043	389 (94.9)	649 (94.3)	0.975	1038 (94.2)
Ampicillin	494 (91.1)	520 (93.5)	0.138	383 (93.4)	631 (91.7)	0.305	1014 (92.5)
Ciprofloxacin	36 (6.6)	62 (11.2)	0.009^b^	29 (7.1)	65 (9.5)	0.174	94 (8.4)
Streptomycin	515 (95)	535 (96.2)	0.329	395 (96.3)	655 (95.2)	0.732	1050 (95.8)
Nalidixic Acid	251 (46.3)	289 (52.1)	0.06	220 (53.7)	320 (46.5)	0.022^b^	540 (48.8)
Chloramphenicol	293 (54.1)	283 (50.9)	0.295	257 (62.7)	319 (46.4)	0.001^b^	576 (52.4)

^a^p values were calculated using the Pearson’s chi-square test.

^b^ p value of less than 0.05 was significant

### Distribution of integron and integron-borne cassettes in *E*. *coli* strains

Of 1098 different isolates from the 134 mother and child pairs screened for classes 1 and 2 integron cassettes, an amplicon was obtained from 410 (37.3%) isolates. Of the 410 isolates, 203 (36.5%) were obtained from the mothers and 207 (38.2%) from children. In all, class 1 integron cassettes alone were amplified from 340 (31%) isolates, class 2 alone from 44 (4%) and class 1 and class 2 integron cassettes were amplified from 26 (2,4%). Integron cassette regions were more commonly amplified from antibiotic-resistant isolates compared to susceptible ones, particularly for trimethoprim, nalidixic acid and chloramphenicol (p ≤ 0.05) (Table 1).

All the isolates were susceptibility tested against eight antimicrobial agents belonging to six classes (folate inhibitors, quinolones, aminoglycosides, penicillins, tetracyclines, and phenicols). Of the 410 integron-positive *E*. *coli* isolates, 404 (98.5%) were resistant to three or more classes of antimicrobial agents. A multidrug resistant phenotype, that is resistance to three or more classes, was significantly more commonly found among integron-positive isolates compared with integron-negative isolates (p<0.05) (Table 2). Isolates from which integrons were identified were commonly resistant to all six classes of antimicrobials (Table 2 and S3 Table). As many as 136 (34%) integron-positive strains were resistant to all agents tested, but ciprofloxacin; even though cassettes encoding resistance to classes other than trimethoprim and the aminoglycosides were rarely or never encountered (the class 1 integron has a sulphonamide resistance cassette at its 3’ conserved end).

**Table 2:** Frequency of multidrug resistance among faecal *Escherichia coli* isolates.

Number of Antimicrobial categories (n = 6)	Number of resistant isolates (n = 1098)	Integron positive isolates (n = 410)	Integron negative isolates (n = 688)	P-value^a^
0	0(0)	0(0)	0(0)	ND
1	3(0.3)	0(0)	3(0.4)	0.297
2	27(2.5)	6(1.5)	21(3.1)	0.149
≥3	1068(97.3)	404(98.5)	664(96.5)	0.0476^b^

^a^p values were calculated using the Pearson’s chi-square test.

^b^ p value of less than 0.05 was significant

### Antibiotic resistance gene cassette content of identified integrons

Ten different gene cassette arrays were found within the integron positive isolates. These consisted of eight varieties in class 1 integrons and two in class 2 integrons (Table 3). In all, three streptomycin and spectinomycin resistance genes (*aadA1*/*A2*/*A5*), six trimethoprim resistance genes (*dfrA1*/*5*/*7*/*15*/*17*/*12*), one gentamicin/tobramycin/kanamycin resistance gene (*aadB*), one streptothricin resistance gene (*satA*) and one open reading frame of unknown function (orfF) were detected.

**Table 3:** Number of integrons and different cassette arrays occurring in faecal *Escherichia coli* isolated from mother-child pairs.

Integron and Cassette Arrays	No. (%) of Isolates		
	Child (n = 542)	Mother (n = 556)	Total (n = 1098)
**Class 1 Integron:**			
One cassette			
*aadA1*	42 (7.7)	54 (9.7)	96 (8.7)
*dfrA7*	33 (6.1)	42 (7.6)	75 (6.8)
*dfrA5*	30 (5.5)	29 (5.2)	59 (5.4)
*dfrA15*	4 (0.7)	6 (1.1)	10 (0.9)
*dfrA17-aadA5*	9 (1.7)	3 (0.5)	12 (1.1)
*dfrA1-aadA1*	39 (7.2)	28 (5.0)	67 (6.1)
*dfrA12-orfF-aadA2*	7 (1.3)	4 (0.7)	11 (1.0)
Two cassette			
*aadA1*, *dfrA1-aadA1*	3 (0.6)	1 (0.2)	4 (0.4)
*dfrA1-aadA1*, *aadB*	2 (0.4)	0 (0)	2 (0.2)
*dfrA15*, *aadA1*	1 (0.2)	0 (0)	1 (0.1)
*dfrA5*, *aadA1*	1 (0.2)	0 (0)	1 (0.1)
*dfrA5*, *dfrA12-orfF-aadA2*	1 (0.2)	1 (0.2)	2 (0.2)
**Total Class 1 Integron**	**168 (31.0)**	**172 (30.9)**	**340 (31.0)**
**Class 2 Integron:**			
*dfrA1-sat1-aadA1*	15 (2.8)	16 (2.9)	31 (2.8)
*dfrA1-sat1*	5 (0.9)	8 (1.4)	13 (1.2)
**Total Class 2 Integron**	**20 (3.7)**	**24 (4.3)**	**44 (4.0)**
Class 1 and 2 Integron:			
*aadA1*, *dfrA1-sat1*	2 (0.4)	1 (0.2)	3 (0.3)
*aadA1*, *dfrA1-sat1-aadA1*	2 (0.4)	1 (0.2)	3 (0.3)
*dfrA12-orfF-aadA2*, *dfrA1-sat1-aadA1*	6 (1.1)	0 (0)	6 (0.5)
*dfrA1-aadA1*, *dfrA1-sat1*	1 (0.2)	0 (0)	1 (0.1)
*dfrA1-aadA1*, *dfrA1-sat1-aadA1*	3 (0.6)	2 (0.4)	5 (0.5)
*dfrA17-aadA5*, *dfrA1-sat1*	1 (0.2)	1 (0.2)	2 (0.2)
*dfrA5*, *dfrA1-sat1*	1 (0.2)	0 (0)	1 (0.1)
*dfrA5*, *dfrA1-sat1-aadA1*	1 (0.2)	0 (0)	1 (0.1)
*dfrA7*, *dfrA1-sat1*	1 (0.2)	0 (0)	1 (0.1)
*dfrA7*, *dfrA1-sat1-aadA1*	1 (0.2)	1 (0.2)	2 (0.2)
*aadA1*, *dfrA1-aadA1*, *dfrA1-Sat1-aadA1*	0 (0)	1 (0.2)	1 (0.1)
**Total Class 1 and 2 Integrons**	**19(3.5)**	**7 (1.3)**	**26 (2.4)**
**Total**	**207 (38.2)**	**203 (36.5)**	**410 (37.3)**

The commonest cassette detected in the class 1 integron context was *aadA1* (n = 96, 8.7%). This was followed by *dfrA7* (n = 75, 6.8%), *dfrA1-aadA1* (n = 67, 6.1%), *dfrA5* (n = 59, 5.4%), *dfrA17-aadA5* (n = 12, 1.1%), *dfrA12-orf-aadA2* (n = 11, 1%) and *dfrA15* (n = 10, 0.9%). Collectively, these seven class 1 cassette combinations represented 330 (97.1%) of all those detected. Only two cassette combinations, *dfrA1-sat1-aadA1* (n = 31, 2.8%) and *dfrA1-sat1* (n = 13, 1.2%) were found in the class 2 integron context. Two cassette arrays were detected in 10 (0.9%) isolates that harboured class 1 integrons while three arrays (*aadA1*, *dfrA1-aadA1*, *dfrA1-Sat1-aadA1*) were detected in one isolate that harboured class 1 and 2 integron.

### Plasmid replicon types and integrons

**Fig 1:**
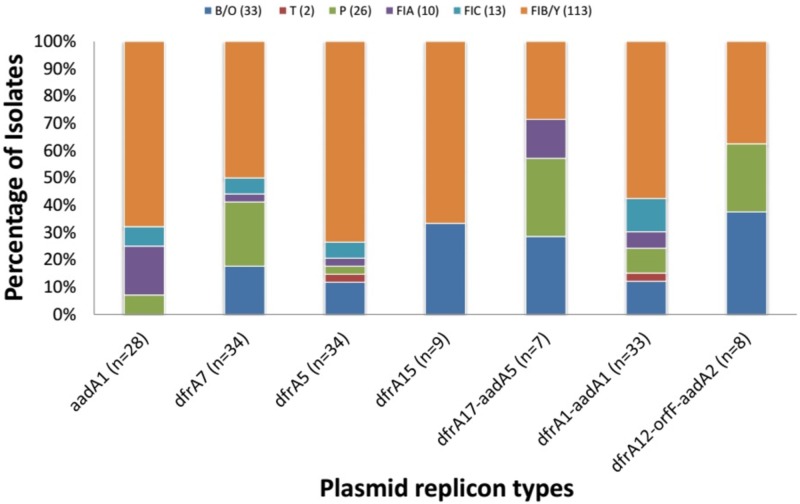
Plasmid replicons types detected in strains harboring only one class 1 integron cassette combination and only one replicon marker site.

Plasmid replicons belonging to all incompatibility groups sought except A, C, K, B and W were identified among integron-containing strains. (The protocol we used does not delineate IncY from the more common IncFIB replicon). As shown in supplementary Table 4, the majority of the integron containing isolates (n = 330, 80.5%) carried one or more of ten different plasmid-replicon-types and at least 133 carried more than one type. IncFIB/Y (n = 169; 41.2%) was the most frequently detected plasmid replicon in the isolates (S4 Table). Pearson regressions did not reveal any association between integron cassette combinations and specific plasmid replicons, pointing to a role for plasmids other than those sought in this study and/or transposons in disseminating integrons (Fig 1).

**Table 4:** Integrons associated with plasmid replicon types in mother and child pairs isolates.

Integrons	Mother only	Child only	Mother-child Pair	Plasmid replicons in paired subjects
**Class 1**				
*aadA1*	54 (26.6)	42 (20.3)	6	FIB/Y, FIA,
*dfrA7*	42 (20.7)	33 (15.9)	9	FIB/Y, P, FIA
*dfrA5*	29 (14.3)	30 (14.5)	6	FIB/Y
*dfrA15*	6 (3)	4 (1.9)	1	None
*dfrA17-aadA5*	3 (1.5)	9 (4.3)	2	FIB/Y
*dfrA1-aadA1*	28 (13.8)	39 (18.8)	7	FIB/Y
*dfrA12-orfF-aadA2*	4 (2.0)	7 (3.4)	1	None
*aadB*	0 (0)	2 (1.0)	0	None
**Class 2**				
*dfrA1-sat1-aadA1*	16 (7.9)	15 (7.2)	2	FIB/Y
*dfrA1-sat1*	8 (3.9)	5 (2.4)	0	None

In 34 instances, the same integron cassette array was detected in at least one *E*. *coli* isolate from a child as well as an isolate from that child’s mother (Table 4), leading us to hypothesize transmission of integron-bearing strains and/or mobile elements within families.

### Diversity among strains bearing the *dfrA5* cassette

We have previously identified a widely disseminated transposon as being responsible for disseminating integron-borne *dfrA7* cassettes among commensal *E*. *coli* isolates in Nigeria and elsewhere in Africa [21]. *dfrA7* was highly prevalent in the current study and associated with a wide variety of plasmid replicons (S4 Table). A single *dfrA5* cassette was the second most common single *dfr* cassette in this study and has been associated with successful invasive non-typhoidal *Salmonella* lineages across Africa [31]. As the context for this cassette among commensals remains unknown, we used a simple PCR marker to define one chromosomal feature and one plasmid feature in each of the strains *dfrA5* isolates obtained in this study to determine whether clonal expansion and/or successful plasmids showed associations with the presence of *dfrA5*.

**Fig 2:**
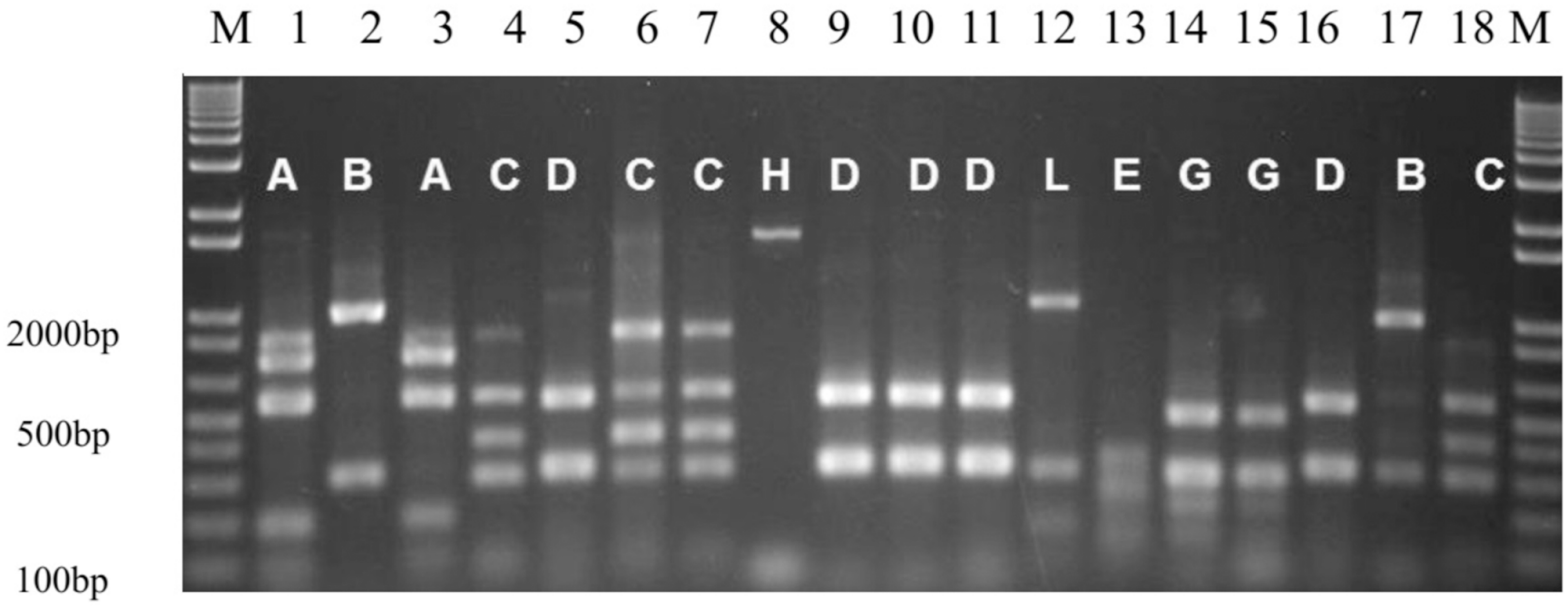
PCR-RFLP *RsaI* restriction pattern types of amplified *fliC* genes of *dfrA5* containing isolates. Lanes 1–20: 1kb+ DNA ladder; 042, c62b, m63b, m103e, c141d, c103d, c102a, m103e, m110a, m110b, m110d, m112a, c118c, c132a, c132b, c141a, c163b, m174e, 1kb+ DNA ladder. c = Child: m = Mother.

**Fig 3:**
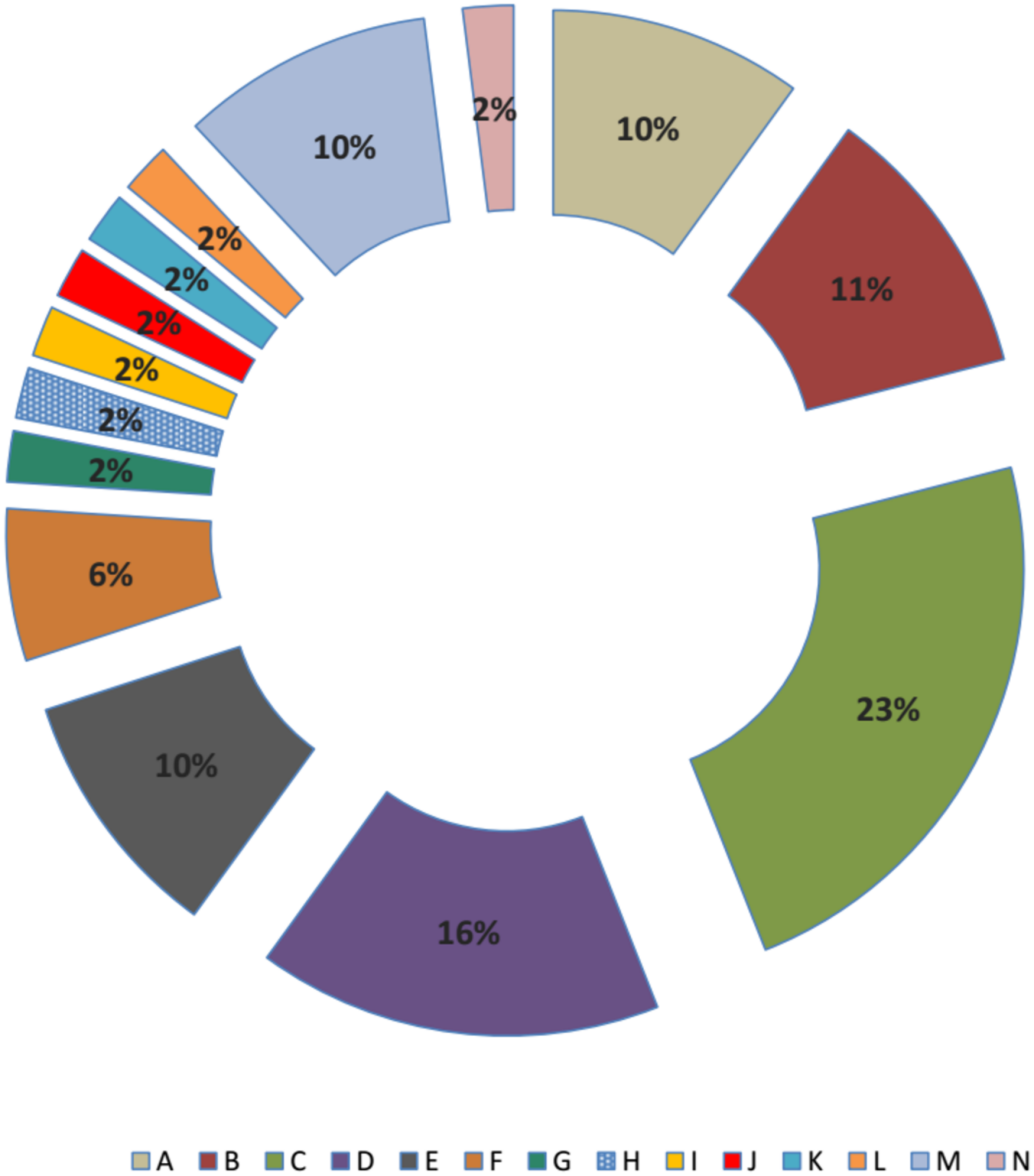
*fliC*-RFLP of *dfrA5* containing isolates (N = 59).

As shown in Figs 2 and 3, fourteen flagellin types were identified and arbitrarily labelled A-N. Type C (23%) was predominantly identified followed by type E (16%) and type B (11%). Multiple distinct isolates with the *dfrA5* cassette were identified in the same host in six instances. In three of those instances, (pairs 246, 250 and 289) the same *fli*C allele was not identified in the mother and the infant whereas in the other three (pairs 130, 174 and 195), identical *fliC* allele although no single allele type was seen in more than one pair. Two of the types seen in pairs were the most common overall (C and E). Of interest is that for four of the six pairs with *dfrA5*-positive isolates, isolates from both the mother and the child carried FIB/Y replicons in four cases and this replicon represents 42.4% of *dfrA5* isolates overall (S5 Table).

### Resistant pandemic lineage in the fecal reservoir of mothers and infants

Fourteen strains out of 1098 *E*. *coli* strains isolated from mother and child pairs carried *dfrA17-aadA5* cassette that is normally found in clonal group A (CGA), a multi-resistant *E*. *coli* belonging to multilocus sequence type (MLST) 69 [32]. These strains comprised of 12 strains with only *dfrA17-aadA5* cassette and two strains with a *dfrA17-aadA5* cassette and *dfrA1-sat1*, a class 2 Integron cassettes (Table 3). As CGA possess a chromosomally located *dfrA17-aadA5*-bearing integron, we sought to determine whether any of the 14 *dfrA17-aadA5*-positive strains identified in this study belong to this clone. CGA strains carry an H18 flagellin (pattern A in our study) and belong to the MLST type 69 whose allele profile includes fumC69. We identified the *fliC*, fumC and recA allele types for the *dfrA17-aadA5*-positive strains. As shown in Table 5, nine isolates from five different individuals carried the CGA-associated H18 *fliC* allele, *fumC*35, and *recA*4. Complete MLST according to the Wirth *et al* [26] scheme confirmed that all nine strains belong to ST69. In one instance, independent *dfrA17-aadA5*-positive isolates were obtained from a mother and her infant.

**Table 5:** Plasmid replicon, *fliC* restriction types and sequence types of *dfrA17-aadA5* isolates.

Strain	Source	Age (months)	fliC RFLP profile	Flagellin *fliC* types	fumC allele	recA allele	Sequence type	Plasmid replicons
C115d	Child	15 mth	A	H18	35	4	69	P
C115e	Child	15 mth	A	H18	35	4	69	P
C139a	Child	11 mth	A	H18	35	4	69	FIA
C139b	Child	11 mth	A	H18	35	4	69	B/O
C139d	Child	11 mth	A	H18	35	4	69	B/O+FIB/Y
C139e	Child	11 mth	A	H18	35	4	69	B/O+FIB/Y
M139b	Mother		A	H18	35	4	69	FIB/Y
C102d	Child	18 mth	B	H30	7	2	not determined	B/O
M102a	Mother		B	H30	53	4	not determined	P+FIB/Y
M102c	Mother		B	H30			not determined	B/O
C228d	Child	12 mth	A	H18	35	4	69	FIB/Y
C78a	Child	12 mth	B	H30			not determined	B/O+FIB/Y
C163b	Child	5 mth	C	H25	4	6	not determined	FIA+FIB/Y
M229a	Mother		A	H18	35	4	69	NIL
SEQ102	CGA control		A	H18	35	4	69	Unknown

C = child, M = mother, SEQ102 = Sequence 102, CGA = clonal group A, mth = month, H = flagella antigen

## Discussion

This study examined integron cassette content in faecal *E*. *coli* from children with diarrhea and their apparently healthy mothers. Integrons are gene exchange systems and are known to play a significant role in the acquisition and dissemination of antimicrobial resistance genes and to be selected by antimicrobial pressure [6]. While our study had the limitation of only detecting cassette regions under 4 Kb in size that had intact 5’ and 3’ ends, we still amplified integron cassettes from as many as 37.3% of isolates. Our detection of 340 (31%) class 1 integron containing strains and 44(4%) class 2 integron containing strains is comparable to data from studies using a similar methodology, which reported a prevalence of integrons to be above 30% [21,33,34,35,36]. Integrons are clearly widespread in faecal *E*. *coli* in this environment and may be responsible for high rates of resistance observed in this study. This is true for trimethoprim where a significant association between the presence of resistance and amplification of integron cassettes was seen and for which eight different cassette arrays including a resistance-conferring *dfrA* gene were detected. The preponderance of the cassette arrays with a *dfrA* gene may be associated with the recent and current intensive use of trimethoprim in many common infections in the study environment as well as to prevent opportunistic infections in HIV-positive patients [21,37,38,39]. Resistance to chloramphenicol and nalidixic acid was also associated with the presence of integrons even though cassettes conferring resistance to these agents were not recovered. This points to integrons as markers of multiresistant strains and the potential that integrons are physically linked to other resistance genes.

Unlike class 2 integrons, the diversity of class 1 integron cassettes/cassette combinations is low when compared with studies performed elsewhere [40, 41]. The low diversity of class 1 cassettes led us to hypothesize that a few strains or mobile elements may be disseminating class 1 integron-mediated resistance. Integrons are not themselves mobile but can be rapidly disseminated when contained within mobile elements such as plasmids. We detected known plasmid replicons in a large number (81.7%) of integron containing isolates examined. In particular, IncF plasmids (predominantly IncFIB/Y replicons but also IncFIA and IncFIC) were commonly found in association with the integron-borne cassettes identified. IncF plasmids represent one of the most prevalent incompatibility types and have been identified worldwide in Enterobacteriaceae from different origins and sources [42,43]. These plasmids appear to significantly contribute to the dissemination of antibiotic resistance in Enterobacteriaceae and some have been associated with specific genes conferring resistance to β-lactams, quinolones, and aminoglycosides [42,44,45,46]. Therefore the preponderance of IncFIB in integron containing isolates may indicate its contribution to the dissemination of integron-borne cassettes in this environment. The study also points to a significant prevalence of IncO plasmids, which have not previously been described from Nigeria.

Of all the cassettes detected in integrons, *aadA1* (n = 96, 23.4%) which encodes resistance to spectinomycin/streptomycin was the commonest. This is in line with various reports of high prevalence of *aadA1* among integron-positive isolates in the literature [20,47]. This was followed by *dfrA7* (n = 75, 18.3%), a cassette that has also been reported worldwide as a commonly identified cassette combination in integrons [48,49,50,51]. In our previous research, focused on different populations and strains, we demonstrated that a single *dfrA7* cassette is the predominant amplifiable cassette combination from fecal *E*. *coli* in Nigeria and elsewhere in Africa [21]. This was also the case in this study. Previously, we found that a widely disseminated transposon accounted for the high frequency of this particular allele [21]. While there were seven other *dfr*-containing configurations, the second most common one, a single d*frA5* cassette was also greatly over-represented in the study. Kingsley *et al* [51] and Okoro *et* al [31] have reported this cassette in *Salmonella enterica* Typhimurium in the context of a transposon that is similar to the one we found associated with *dfrA7*. S. Typhimurium strains carrying this transposon represent a successful lineage that has expanded at multiple sites on the African continent. We wanted to know whether overrepresentation of *dfrA5* in this study was due to clonal expansion of one or a few *E*. *coli* lineages or mobility of the cassette through the commensal flora by virtue of a transposon or other mobile element. Flagellin typing of the 59 isolates that carried a single *dfrA5* cassette revealed that while some flagellin types were very common, none represented more than 23% of the set and there were 14 flagellin types overall. As each flagellin type is seen in multiple *E*. *coli* lineages, it is unlikely that a significant expansion of any *dfrA5*-bearing clone has occurred. Dissemination of *dfrA5* across the commensal flora could be due to a successful plasmid or a more modular element such as a transposon that can associate with different plasmids or the chromosome [52]. In the case of the former, a single plasmid marker would be overrepresented in the set. However PCR screening for eleven plasmid replicons identified 49 among *dfrA5* strains and different replicons were associated with different flagellin types. Altogether the data point to diverse strain backgrounds and possible over-representation of FIB/Y plasmids in integron bearing strains but little indication of clonal expansion of a distinct *dfrA5*-bearing lineage. The dissemination of *dfrA5* could also be due to a more modular mechanism potentially similar to what we have previously seen for *dfrA7*. Venturini *et al*. [52] recently reported a role for IS26 elements in disseminating *dfrA5*, which could very well be under play in our own study environment.

In addition to pointing to flexible contexts for more common integron arrays, this study also provided a little insight into circulation of a successful pandemic lineage, the *dfrA17-aadA5*-bearing ST69 CGA in the faecal microflora. Multi-resistant *E*. *coli* belonging to multilocus sequence type (MLST) 69 have been termed CGA and implicated in invasive infections in different parts of the world, including Nigeria [32]. The epidemiology of CGA in Nigeria is understudied since pandemic lineages are rarely sought here. As CGA possess a chromosomally located *dfrA17-aadA5*-bearing integron, we sought to determine whether any of the 14 *dfrA17-aadA5*-positive strains identified in this study belong to this clone. Likely members of this lineage represented a small minority of the integron-bearing strains detected in this study. Verified ST69 strains were detected in 5 (1.9%) of 264 individuals including both members of one mother-infant pair. Our data suggest that CGA may be shared among individuals in the same household and its presence may facilitate the dissemination of *dfrA17-aadA5* cassette in this environment.

## Conclusions

In conclusion, this study reveals the presence of integrons in fecal *E*. *coli* isolated from apparently healthy mothers and their sick children in Nigeria. The identified integrons contain antibiotic-resistant gene cassettes and while the variety of cassette combinations is very limited, the backgrounds of strains carrying these elements and the repertoire of plasmids associated with them are reasonably diverse. The detection of different replicons in association with different flagellin types in *dfrA5* strains, points to a successful plasmid or a more modular element that is borne on plasmids or located on the chromosome for its dissemination. In view of the findings of this study, there is a need for improved surveillance, which can provide information for the persistence and mobility of resistance genes between the community and clinical settings.

## Supporting information

S1 Table Primers for identification of class 1 and class 2 integrons. (DOCX)

S2 Table Multi-locus sequence typing primers. (DOCX)

S3 Table Resistance phenotypes *of Escherichia coli* isolates and their associated integron cassettes. (DOCX)

S4 Table Plasmid replicon types associated with integrons in *Escherichia coli* isolates. (DOCX)

S5 Table Antimicrobial resistant *Escherichia coli* isolates containing class1 integrons with *dfrA5* isolates cassettes. (DOCX)
