# Correction: Classes 1 and 2 integrons in faecal *Escherichia coli* strains isolated from mother-child pairs in Nigeria

**DOI:** 10.1371/journal.pone.0197202

**Published:** 2018-05-07

**Authors:** Babatunde W. Odetoyin, Amy S. Labar, Adebayo Lamikanra, Aaron O. Aboderin, Iruka N. Okeke

There are errors in the Author Contributions. The correct contributions are: Investigation: INO AOA ASL BWO. Supervision: INO AOA. Writing–review & editing: BWO INO AOA ASL AL. The second, third and fourth authors, Amy S. Labar, Adebayo Lamikanra and Aaron O. Aboderin, should not have been attributed equal contribution to this work.
