# Supplementary material for: Classes 1 and 2 integrons in faecal Escherichia coli strains isolated from mother-child pairs in Nigeria
Source: PLoS One. 2017 Aug 22;12(8):e0183383. doi: 10.1371/journal.pone.0183383 (PMC5568733; doi:10.1371/journal.pone.0183383)
Supplement: S1 Table — (DOCX) [file pone.0183383.s001.docx]

**S1 Table. Primers for Identification of class 1 and class 2 Integrons**

| Target | **Primers** | **Sequence (5' to 3')** |
| --- | --- | --- |
| Class 1 Integron | Lévesque5CS | GGC ATC CAA GCA GCA AG |
|  | Lévesque3CS | AAG CAG ACT TGA CCT GA |
| Class 2 Integron | Whitehep 74 | CGG GAT CCC GGA CGG CAT GCA CGA TTT GTA |
|  | Whitehep 51 | GAT GCC ATC GCA AGT ACG AG |
