# Supplementary material for: Classes 1 and 2 integrons in faecal Escherichia coli strains isolated from mother-child pairs in Nigeria
Source: PLoS One. 2017 Aug 22;12(8):e0183383. doi: 10.1371/journal.pone.0183383 (PMC5568733; doi:10.1371/journal.pone.0183383)
Supplement: S2 Table — (DOCX) [file pone.0183383.s002.docx]

**S2 Table: Multi-locus sequence typing primers**

| **Target gene** | Primer Sequence | | **Amplicon size (bp)** |
| --- | --- | --- | --- |
|  | Direction | Sequence (5’ to 3’) |  |
| *adk* | F | ATTCTGCTTGGCGCTCCGGG | 536 |
|  | R | CCGTCAACTTTCGCGTATTT |  |
| *fumC* | F | TCACAGGTCGCCAGCGCTTC | 469 |
|  | R | GTACGCAGCGAAAAAGATTC |  |
| *gyrB* | F | TCGGCGACACGGATGACGGC | 460 |
|  | R | ATCAGGCCTTCACGCGCATC |  |
| *icd* | F | ATGGAAAGTAAAGTAGTTGTTCCGGCACA | 518 |
|  | R | GGACGCAGCAGGATCTGTT |  |
| *mdh* | F | ATGAAAGTCGCAGTCCTCGGCGCTGCTGGCGG | 452 |
|  | R | TTAACGAACTCCTGCCCCAGAGCGATATCTTTCTT |  |
| *purA* | F | CGCGCTGATGAAAGAGATGA | 478 |
|  | R | CATACGGTAAGCCACGCAGA |  |
| *recA* | F | CGCATTCGCTTTACCCTGACC | 510 |
|  | R | TCGTCGAAATCTACGGACCGGA |  |

*adk-* adenylate kinase, *fumC*- fumarate hydratase, *gyrB-* gyrase B, *icd-* isocitrate dehydrogenase, *mdh*- malate dehydrogenase *purA-* adenylosuccinate synthetase, *recA*- recombinase A
