# Supplementary material for: Classes 1 and 2 integrons in faecal Escherichia coli strains isolated from mother-child pairs in Nigeria
Source: PLoS One. 2017 Aug 22;12(8):e0183383. doi: 10.1371/journal.pone.0183383 (PMC5568733; doi:10.1371/journal.pone.0183383)
Supplement: S3 Table — (DOCX) [file pone.0183383.s003.docx]

| **No of Strain**  **S3 Table: Resistance phenotypes *of Escherichia coli* isolates and their associated integron cassettes** | **Resistance Phenotypes** | **Class 1** | **Class 2** |
| --- | --- | --- | --- |
| 18 | Tet, **Trim**, Sul, Amp, Cip, **Str**, Nal, chl | *aadA1, dfrA7, dfrA1-aadA, dfrA12-orF-aadA2* | *dfrA1-sat1-aadA1* |
| 4 | Tet, **Trim**, Sul, Amp, Cip, Str, Nal | *dfrA1-aadA1, aadA1* |  |
| 1 | Tet, **Trim**, Sul, Amp, Cip, Str | *dfrA1-aadA1* |  |
| 136 | Tet, **Trim**, Sul, Amp, **Str**, Nal, chl | *aadA1, dfrA15, dfrA7, dfrA1-aadA1, dfrA5, dfrA12-orF-aadA2* | *dfrA1-sat1-aadA1, dfrA1-sat1* |
| 1 | **Trim**, Sul, Amp, **Str**, Nal, chl | *aadA1* |  |
| 2 | Sul, Amp, **Str**, Nal, chl | *dfrA5, dfrA7* |  |
| 2 | Tet, **Trim**, Sul, **Str**, Nal, chl | *aadA1, dfrA1-aadA1* |  |
| 33 | Tet, **Trim**, Sul, Amp, **Str**, Nal | *aadA1, dfrA17-aadA5, dfrA1-aadA1, dfrA5, dfrA7, dfrA12-orF-aadA2* | *dfrA1-Sat1-aadA1, dfrA1-sat1* |
| 78 | Tet, **Trim**, Sul, Amp, **Str**, chl | *aadA1, dfrA5, dfrA7, dfrA15, dfrA1-aadA1, dfrA17-aadA5, aadB*, *dfrA12-orF-aadA2* | *dfrA1-Sat1-aadA1, dfrA1-sat1* |
| 65 | Tet, **Trim**, Sul, Amp, **Str** | *aadA1, dfrA15, dfrA12-orF-aadA2, dfrA1-aadA1, dfrA5, dfrA7* | *dfrA1-Sat1-aadA1, dfrA1-sat1* |
| 2 | Tet, **Trim**, Sul, Amp, Nal, chl | *dfrA5, dfrA7* |  |
| 2 | Tet, **Trim**, Sul, Amp, chl | *aadA1* |  |
| 4 | Tet, **Trim**, Sul, Amp | *aadA1, dfrA1-aadA1* | *dfrA1-sat1* |
| 7 | Tet, **Trim**, Sul, Strep, chl | *dfrA1-aadA1, aadA1, dfrA12-orF-aadA2* |  |
| 1 | Tet, **Trim**, Sul, Cip, Str, Nal, chl | *dfrA5* |  |
| 1 | Tet, **Trim**, Amp, Nal, chl | *aadA1* |  |
| 2 | Tet, **Trim**, Amp, **Str**, chl | *dfrA7* | *dfrA1-sat1-aadA1* |
| 4 | **Trim**, Sul, Amp, **Str**, chl | *dfrA1-aadA1, aadA1, dfrA5* | *dfrA1-sat1-aadA1* |
| 2 | Tet, **Trim**, Amp, **Str** | *dfrA7, dfrA1-aadA1* |  |
| 7 | Tet, **Trim**, Sul, **Str** | *aadA1, dfrA5, dfrA7, dfrA1-aadA1* | *dfrA1-Sat1-aadA1* |
| 3 | Tet, Sul, Amp, **Str** | *dfrA1-aadA1, aadA1, dfrA12-orF-aadA2* | *dfrA1-sat1-aadA1* |
| 2 | Sul, Amp, **Str** | *dfrA17-aadA5, dfrA5* |  |
| 2 | **Trim**, Sul, Amp, **Str** | *dfrA7* |  |
| 1 | Tet, Amp | *dfrA7* |  |
| 3 | Tet, Sul, **Str** | *aadA1, dfrA1-aadA1* |  |
| 1 | Tet, Cip, Str | *dfrA5* |  |
| 1 | Tet, Sul | *aadA1* |  |
| 3 | Tet, Sul, **Str**, Nal | *aadA1, dfrA1-aadA1* | *dfrA1-Sat1* |
| 1 | **Trim**, Sul, Nal |  | *dfrA1-Sat1-aadA1* |
| 1 | Tet, Sul, Amp, **Str**, Nal | *aadA1,* | *dfrA1-sat1-aadA1* |
| 1 | Tet, **Trim**, Amp, Cip, **Str**, Nal |  | *dfrA1-sat1-aadA1* |
| 1 | Tet, Amp, Nal | *dfrA7* |  |
| 1 | Amp, Cip | *aadA1* |  |
| 2 | Amp, **Str**, Nal | *aadA1, dfrA7* |  |
| 5 | **Trim**, Sulp, Amp, **Str**, Nal | *dfrA5, aadA1, dfrA17-aadA5* |  |
