# Supplementary material for: Classes 1 and 2 integrons in faecal Escherichia coli strains isolated from mother-child pairs in Nigeria
Source: PLoS One. 2017 Aug 22;12(8):e0183383. doi: 10.1371/journal.pone.0183383 (PMC5568733; doi:10.1371/journal.pone.0183383)
Supplement: S4 Table — (DOCX) [file pone.0183383.s004.docx]

| **Plasmid Replicon** | ***aadA1*** | ***dfrA7*** | ***dfrA5*** | ***dfrA15*** | ***dfrA17-aadA5*** | ***dfrA1-aadA1*** | ***dfrA12-orfF-aadA2*** | ***aadA1, dfrA1-aadA1*** | ***dfrA1-aadA1, aadB*** | ***dfrA15, aadA1*** | ***dfrA5, aadA1*** | ***dfrA5, dfrA12-orfF-aadA2*** | ***dfrA1-sat1-aadA1*** | ***dfrA1-sat1*** | ***aadA1, dfrA1-sat1*** | ***aadA1, dfrA1-sat1-aadA1*** | ***dfrA12-orfF-aadA2, dfrA1-sat1-aadA1*** | ***dfrA1-aadA1, dfrA1-sat1*** | ***dfrA1-aadA1, dfrA1-sat1-aadA1*** | ***dfrA17-aadA5, dfrA1-sat1*** | ***dfrA5, dfrA1-sat1*** | ***dfrA5, dfrA1-sat1-aadA1*** | ***dfrA7, dfrA1-sat1*** | ***dfrA7, dfrA1-sat1 aadA1*** | ***aadA1, dfrA1-aadA1, dfrA1-Sat1-aadA1*** |
| --- | --- | --- | --- | --- | --- | --- | --- | --- | --- | --- | --- | --- | --- | --- | --- | --- | --- | --- | --- | --- | --- | --- | --- | --- | --- |
| **One plasmid (197)** | 28 | 34 | 34 | 9 | 7 | 33 | 8 | 2 | 2 | 1 | 0 | 2 | 16 | 8 | 2 | 2 | 3 | 0 | 4 | 1 | 0 | 0 | 1 | 0 | 0 |
| K/B (0) | 0 | 0 | 0 | 0 | 0 | 0 | 0 | 0 | 0 | 0 | 0 | 0 | 0 | 0 | 0 | 0 | 0 | 0 | 0 | 0 | 0 | 0 | 0 | 0 | 0 |
| O (33) | 0 | 6 | 4 | 3 | 2 | 4 | 3 | 1 | 0 | 0 | 0 | 1 | 1 | 1 | 1 | 1 | 1 | 0 | 3 | 1 | 0 | 0 | 0 | 0 | 0 |
| T (2) | 0 | 0 | 1 | 0 | 0 | 1 | 0 | 0 | 0 | 0 | 0 | 0 | 0 | 0 | 0 | 0 | 0 | 0 | 0 | 0 | 0 | 0 | 0 | 0 | 0 |
| A/C (0) | 0 | 0 | 0 | 0 | 0 | 0 | 0 | 0 | 0 | 0 | 0 | 0 | 0 | 0 | 0 | 0 | 0 | 0 | 0 | 0 | 0 | 0 | 0 | 0 | 0 |
| P (26) | 2 | 8 | 1 | 0 | 2 | 3 | 2 | 1 | 0 | 0 | 0 | 0 | 2 | 3 | 0 | 1 | 0 | 0 | 0 | 0 | 0 | 0 | 1 | 0 | 0 |
| W (0) | 0 | 0 | 0 | 0 | 0 | 0 | 0 | 0 | 0 | 0 | 0 | 0 | 0 | 0 | 0 | 0 | 0 | 0 | 0 | 0 | 0 | 0 | 0 | 0 | 0 |
| FIA (10) | 5 | 1 | 1 | 0 | 1 | 2 | 0 | 0 | 0 | 0 | 0 | 0 | 0 | 0 | 0 | 0 | 0 | 0 | 0 | 0 | 0 | 0 | 0 | 0 | 0 |
| FIC (13) | 2 | 2 | 2 | 0 | 0 | 4 | 0 | 0 | 0 | 1 | 0 | 1 | 0 | 1 | 0 | 0 | 0 | 0 | 0 | 0 | 0 | 0 | 0 | 0 | 0 |
| FIB/Y (113) | 19 | 17 | 25 | 6 | 2 | 19 | 3 | 0 | 2 | 0 | 0 | 0 | 13 | 3 | 1 | 0 | 2 | 0 | 1 | 0 | 0 | 0 | 0 | 0 | 0 |
| **More than one (133)** | 40 | 26 | 15 | 0 | 4 | 23 | 0 | 2 | 0 | 0 | 0 | 0 | 11 | 4 | 1 | 0 | 1 | 1 | 0 | 1 | 1 | 1 | 0 | 2 | 0 |
| O+P+FIA+FIBY(6) | 2 | 1 | 0 | 0 | 0 | 2 | 0 | 0 | 0 | 0 | 0 | 0 | 1 | 0 | 0 | 0 | 0 | 0 | 0 | 0 | 0 | 0 | 0 | 0 | 0 |
| P+FIA+FIB/Y (21) | 6 | 3 | 2 | 0 | 0 | 4 | 0 | 0 | 0 | 0 | 0 | 0 | 4 | 1 | 0 | 0 | 0 | 0 | 0 | 1 | 0 | 0 | 0 | 0 | 0 |
| O+FIC+FIBY(7) | 4 | 2 | 0 | 0 | 0 | 1 | 0 | 0 | 0 | 0 | 0 | 0 | 0 | 0 | 0 | 0 | 0 | 0 | 0 | 0 | 0 | 0 | 0 | 0 | 0 |
| O+P+FIB/Y (2) | 2 | 0 | 0 | 0 | 0 | 0 | 0 | 0 | 0 | 0 | 0 | 0 | 0 | 0 | 0 | 0 | 0 | 0 | 0 | 0 | 0 | 0 | 0 | 0 | 0 |
| P+W (5) | 0 | 1 | 1 | 0 | 0 | 3 | 0 | 0 | 0 | 0 | 0 | 0 | 0 | 0 | 0 | 0 | 0 | 0 | 0 | 0 | 0 | 0 | 0 | 0 | 0 |
| FIA+FIB/Y (54) | 15 | 14 | 7 | 0 | 1 | 8 | 0 | 1 | 0 | 0 | 0 | 0 | 3 | 2 | 0 | 0 | 0 | 1 | 0 | 0 | 0 | 0 | 0 | 2 | 0 |
| O+P+FIA (7) | 1 | 1 | 1 | 0 | 0 | 1 | 0 | 0 | 0 | 0 | 0 | 0 | 0 | 1 | 1 | 0 | 1 | 0 | 0 | 0 | 0 | 0 | 0 | 0 | 0 |
| K/B+P+W (2) | 0 | 0 | 0 | 0 | 0 | 1 | 0 | 0 | 0 | 0 | 0 | 0 | 1 | 0 | 0 | 0 | 0 | 0 | 0 | 0 | 0 | 0 | 0 | 0 | 0 |
| FIA+O+FIC (2) | 0 | 1 | 0 | 0 | 0 | 0 | 0 | 1 | 0 | 0 | 0 | 0 | 0 | 0 | 0 | 0 | 0 | 0 | 0 | 0 | 0 | 0 | 0 | 0 | 0 |
| K/B+O (2) | 0 | 0 | 2 | 0 | 0 | 0 | 0 | 0 | 0 | 0 | 0 | 0 | 0 | 0 | 0 | 0 | 0 | 0 | 0 | 0 | 0 | 0 | 0 | 0 | 0 |
| O+FIB/Y (23) | 8 | 3 | 2 | 0 | 3 | 3 | 0 | 0 | 0 | 0 | 0 | 0 | 2 | 0 | 0 | 0 | 0 | 0 | 0 | 0 | 1 | 1 | 0 | 0 | 0 |
| P+FIC (2) | 2 | 0 | 0 | 0 | 0 | 0 | 0 | 0 | 0 | 0 | 0 | 0 | 0 | 0 | 0 | 0 | 0 | 0 | 0 | 0 | 0 | 0 | 0 | 0 | 0 |
| **Unknown plasmid (80)** | 28 | 15 | 10 | 1 | 1 | 11 | 3 | 0 | 0 | 0 | 1 | 0 | 4 | 1 | 0 | 1 | 2 | 0 | 1 | 0 | 0 | 0 | 0 | 0 | 1 |
| **Total (410)** | 96 | 75 | 59 | 10 | 12 | 67 | 11 | 4 | 2 | 1 | 1 | 2 | 31 | 13 | 3 | 3 | 6 | 1 | 5 | 2 | 1 | 1 | 1 | 2 | 1 |

**S4 Table: Plasmid replicon types associated with integrons in *Escherichia coli* isolates**
