# Supplementary material for: Classes 1 and 2 integrons in faecal Escherichia coli strains isolated from mother-child pairs in Nigeria
Source: PLoS One. 2017 Aug 22;12(8):e0183383. doi: 10.1371/journal.pone.0183383 (PMC5568733; doi:10.1371/journal.pone.0183383)
Supplement: S5 Table — (DOCX) [file pone.0183383.s005.docx]

**S5 Table: Antimicrobial resistant *Escherichia coli* isolates containing class1 integrons with *dfrA5* isolates cassettes**

| **Strain** | **Host** | **Plasmid** | Resistance Patterns | | | | | | | **cassette content** | | **fliC pattern** | |  |
| --- | --- | --- | --- | --- | --- | --- | --- | --- | --- | --- | --- | --- | --- | --- |
| C39b | Child | B/O+FIA | Tet | Tri | Sul | Amp | Str | Nal |  | | *dfrA5* | | C | |
| C62b | Child | FIA | Tet | Tri | Sul | Amp | Str | Nal | Chlo | | *dfrA5* | | B | |
| M63a | Mother | P | Tet | Tri | Sul | Amp | Str | Nal | Chlo | | *dfrA5* | | A | |
| M103e | Mother | FIB/Y | Tet | Tri | Sul | Amp | Str | Nal | Chlo | | *dfrA5* | | G | |
| M112a | Mother | B/O | Tet | Tri | Sul | Amp | Str | Nal | | | *dfrA5* | | B | |
| M112b | Mother | B/O | Tet | Tri | Sul | Amp | Str | Nal | | | *dfrA5* | | B | |
| M112c | Mother | B/O | Tet | Tri | Sul | Amp | Str | Nal | | | *dfrA5* | | B | |
| M112e | Mother | FIB/Y | Tet | Tri | Sul | Amp | Str | Nal | | | *dfrA5* | | B | |
| C118c | Child | FIB/Y | Sul | Amp | Str |  | | | | | *dfrA5* | | E | |
| C129a | Child | FIB/Y | Tet | Tri | Sul | Amp | Str | Nal | Chlo | | *dfrA5* | | C | |
| C130b | Child | FIB/Y | Tet | Tri | Sul | Amp | Str | Nal | Chlo | | *dfrA5* | | C | |
| C130c | Child | FIB/Y | Tet | Tri | Sul | Cip | Str | Nal | Chlo | | *dfrA5* | | C | |
| C130d | Child | FIB/Y | Tet | Tri | Sul | Amp | Str | Nal | Chlo | | *dfrA5* | | C | |
| M130a | Mother | T | Tet | Tri | Sul | Amp | Str | Nal | Chlo | | *dfrA5* | | L | |
| M130b | Mother | FIC | Tet | Tri | Sul | Amp | Str | Nal | Chlo | | *dfrA5* | | C | |
| M130c | Mother | FIB/Y | Tet | Tri | Sul | Amp | Nal | Chlo |  | | *dfrA5* | | N | |
| M130d | Mother | FIB/Y | Tet | Tri | Sul | Amp | Str | Nal | Chlo | | *dfrA5* | | K | |
| M130e | Mother | FIB/Y | Tet | Tri | Sul | Amp | Str | Nal | Chlo | | *dfrA5* | | C | |
| C134a | Mother | P+FIA | Trim | Sul | Amp | Str | Nal |  | | | *dfrA5* | | M | |
| M137d | Mother | FIB/Y | Tet | Trim | Sul | Amp | Str | Chlo | | | *dfrA5* | | D | |
| M139c | Mother | FIB/Y | Tet | Tri | Sul | Amp | Str | Nal | Chlo | | *dfrA5* | | L | |
| M140d | Mother | FIB/Y | Sul | Amp | Str | Nal | Chlo |  | | | *dfrA5* | | J | |
| C141a | Child | B/O | Tet | Trim | Sul | Amp | Str | Chlo | | | *dfrA5* | | D | |
| C141c | Child | B/O+FIB/Y | Tet | Tri | Sul | Amp | Str | Nal | Chlo | | *dfrA5* | | D | |
| C141e | Child | B/O+FIB/Y | Tet | Trim | Sul | Amp | Str | Chlo |  | | *dfrA5* | | D | |
| C161c | Child | No | Tet | Tri | Sul | Amp | Str | Nal | Chlo | | *dfrA5* | | D | |
| C163c | Child | FIB/Y | Tet | Tri | Sul | Amp | Str | Nal | Chlo | | *dfrA5* | | B | |
| C174b | Child | FIB/Y | Tet | Tri | Sul | Amp | Str | Nal | Chlo | | *dfrA5* | | N | |
| C174c | Child | FIB/Y | Tet | Tri | Sul | Amp | Str | Nal | Chlo | | *dfrA5* | | N | |
| C174d | Child | FIB/Y | Tet | Tri | Sul | Amp | Str | Nal | Chlo | | *dfrA5* | | N | |
| C174e | Child | FIB/Y | Tet | Tri | Sul | Amp | Str | Nal | Chlo | | *dfrA5* | | M | |
| M174b | Mother | FIB/Y | Tet | Tri | Sul | Amp | Str | Nal | Chlo | | *dfrA5* | | N | |
| M174c | Mother | FIB/Y | Tet | Tri | Sul | Amp | Str | Nal | Chlo | | *dfrA5* | | N | |
| M174e | mother | FIB/Y | Tet | Tri | Sul | Amp | Str | Nal | Chlo | | *dfrA5* | | C | |
| C190b | child | FIB/Y | Tet | Trim | Sul | Amp | Str | Chlo | | | *dfrA5* | | A | |
| C190d | child | FIB/Y | Tet | Trim | Sul | Amp | Str | Chlo | | | *dfrA5* | | A | |
| C195e | child | No | Tet | Trim | Sul | Amp | Str | Chlo | | | *dfrA5* | | E | |
| M195d | mother | B/O+K/B | Tet | Trim | Sul | Amp | Str |  | | | *dfrA5* | | E | |
| C198a | child | B/O+K/B | Tet | Trim | Sul | Amp | Str |  | | | *dfrA5* | | E | |
| M201a | mother | Y | Tet | Trim | Sul | Amp | Str | Chlo | | | *dfrA5* | | C | |
| C207c | child | FIA+FIB/Y | Tet | Trim | Amp | Str | Chlo |  | | | *dfrA5* | | I | |
| C209e | child | FIA+FIB/Y | Tet | Trim | Amp | Str | Chlo |  | | | *dfrA5* | | E | |
| M211c | mother | FIC | Tet | Trim | Sul | Amp | Str | Chlo | | | *dfrA5* | | H | |
| C231b | child | No | Tet | Trim | Sul | Str | | | | | *dfrA5* | | M | |
| M228c | mother | No | Tet | Cip | Str |  | | | | | *dfrA5* | | C | |
| C246b | child | FIA+FIB/Y | Tet | Trim | Sul | Amp | Str | Nal |  | | *dfrA5* | | M | |
| M246b | mother | P+W | Tet | Trim | Sul | Amp | Str | Nal |  | | *dfrA5* | | C | |
| C250d | child | FIB/Y+Y | Tet | Trim | Sul | Amp | Str | Nal | Chlo | | *dfrA5* | | A | |
| M250d | mother | FIA+FIB/Y | Tet | Trim | Sul | Amp | Str | Nal | Chlo | | *dfrA5* | | C | |
| M265a | mother | No | Tet | Trim | Sul | Amp | Str | Nal |  | | *dfrA5* | | D | |
| M278e | mother | FIA+FIB | Tet | Trim | Sul | Amp | Str | Nal | Chlo | | *dfrA5* | | E | |
| M285b | mother | No | Tet | Trim | Sul | Amp | Str | Chlo |  | | *dfrA5* | | F | |
| C289e | child | No | Tet | Trim | Sul | Amp | Str | Nal | Chlo | | *dfrA5* | | F | |
| M289c | mother | No | Tet | Trim | Sul | Amp | Str | Chlo |  | | *dfrA5* | | C | |
| C293b | child | FIA+FIB | Trim | Sul | Amp | Str | Chlo |  | | | *dfrA5* | | M | |
| C293c | child | FIA+FIB | Trim | Sul | Amp | Str | Chlo |  | | | *dfrA5* | | M | |
| M312b | mother | No | Tet | Trim | Sul | Amp | Str | Nal | Chlo | | *dfrA5* | | A | |
| C312c | child | No | Tet | Trim | Sul | Amp | Str | Nal | Chlo | | *dfrA5* | | A | |
| C312d | Child | P+FIA+FIB | Tet | Trim | Sul | Amp | Str | Nal | Chlo | | *dfrA5* | | C | |
| 042 | Control | Unknown | Tet | Trim | Sul | Str | | | | | *aadA1* | | A | |

Tet=Tetracycline, Trim=Trimetoprim, Sul=Sulphonamide, Amp=Ampicillin, Str=Streptomycin, Nal=Nalidixic acid, Chlo=Chloramphenicol
